# Incidence of Type II CRISPR1-Cas Systems in *Enterococcus* Is Species-Dependent

**DOI:** 10.1371/journal.pone.0143544

**Published:** 2015-11-24

**Authors:** Casandra Lyons, Nicole Raustad, Mario A. Bustos, Michael Shiaris

**Affiliations:** Biology Department, University of Massachusetts Boston, Boston, Massachusetts, United States of America; University Medical Center Utrecht, NETHERLANDS

## Abstract

CRISPR-Cas systems, which obstruct both viral infection and incorporation of mobile genetic elements by horizontal transfer, are a specific immune response common to prokaryotes. Antiviral protection by CRISPR-Cas comes at a cost, as horizontally-acquired genes may increase fitness and provide rapid adaptation to habitat change. To date, investigations into the prevalence of CRISPR have primarily focused on pathogenic and clinical bacteria, while less is known about CRISPR dynamics in commensal and environmental species. We designed PCR primers and coupled these with DNA sequencing of products to detect and characterize the presence of *cas1*, a universal CRISPR-associated gene and proxy for the Type II CRISPR1-Cas system, in environmental and non-clinical *Enterococcus* isolates. CRISPR1-*cas1* was detected in approximately 33% of the 275 strains examined, and differences in CRISPR1 carriage between species was significant. Incidence of *cas1* in *E*. *hirae* was 73%, nearly three times that of *E*. *faecalis* (23.6%) and 10 times more frequent than in *E*. *durans* (7.1%). Also, this is the first report of CRISPR1 presence in *E*. *durans*, as well as in the plant-associated species *E*. *casseliflavus* and *E*. *sulfureus*. Significant differences in CRISPR1-*cas1* incidence among *Enterococcus* species support the hypothesis that there is a tradeoff between protection and adaptability. The differences in the habitats of enterococcal species may exert varying selective pressure that results in a species-dependent distribution of CRISPR-Cas systems.

## Introduction

Bacteria and Archaea possessing CRISPR-Cas systems trade off horizontally-acquired adaptation to a changing environment for protection against lethal virus infection. CRISPRs are clustered regularly interspaced short palindromic repeats of DNA; Cas refers to CRISPR-associated proteins. Together, they comprise a uniquely prokaryotic multi-step adaptive immune response that provides defense against bacteriophage infection [1]. In the process, incorporation of transmissible genetic elements is interrupted, including plasmids and DNA with potential advantages for the host cell, such as those conferring antibiotic resistance [2]. Briefly, fragments of non-self DNA called protospacers are acquired by Cas proteins, and incorporated as spacers between the DNA repeats of the CRISPR array. These repeat-spacer modules are transcribed and expressed as crRNAs, a small interference-type RNA. If invading nucleic acid has a short sequence with perfect complementarity to the spacer region of the crRNA, a sequence-specific cleavage event is initiated, degrading the foreign nucleic acids [3,4]. CRISPR arrays are widespread among Bacteria and Archaea, in approximately 90% of archaeal and 40% of bacterial genomes examined [5,6]. The diversity of CRISPR systems is extensive. CRISPRs may be broadly divided into those lacking *cas* genes, thus consisting solely of repeat-spacer arrays (also referred to as orphan CRISPRs), and those comprised of both an array and associated functional genes (CRISPR-Cas). CRISPR-Cas systems are further divided into types and subtypes, defined by presence of subtype-specific Cas proteins [7]. Several Cas proteins are considered universal, with orthologs appearing in every active subtype. One of these is Cas1 [7,8]. Encoded by a single gene (*cas1*), the ubiquity of Cas1 makes it a suitable marker for the presence of a potentially active CRISPR-Cas system.

We focused on CRISPR1 systems in the genus *Enterococcus*, a clade of commensal bacteria common to animal and human gut microflora. Enterococci emerged as a cause of multidrug resistant hospital acquired infection in the 1970s, and presently represent one of the most prevalent causes of nosocomial infections in the United States [9]. Two species–*E*. *faecalis* and *E*. *faecium*–are primarily responsible for these infections [10]. They are also the predominant enterococcal human gastrointestinal (GI) commensals [11]. Mobile elements, including plasmids, pathogenicity islands, and antibiotic resistance genes, comprise as much as 25% of the genomes of hospital-adapted lineages of both species [12,13,14]. Palmer and Gilmore (15) showed that multiple drug resistance and incidence of CRISPR-Cas are negatively correlated in *E*. *faecalis* and *E*. *faecium*. That is, their results suggest that there is a tradeoff between acquisition of drug resistance and CRISPR-mediated protection from foreign DNA. Three Type II CRISPRs have been identified in human GI *E*. *faecalis*: two with associated *cas* genes (CRISPR1-Cas and CRISPR3-Cas) and one orphan repeat-spacer array (CRISPR2) [15]. CRISPR2 is present in 95% of *E*. *faecalis* isolates; as many as half of these strains contain CRISPR1-Cas, and CRISPR3-Cas has been detected in four *E*. *faecalis* genomes to date [15,16]. This suggests that species under different selective pressures may vary significantly in their incidence of CRISPR.

Several studies have investigated CRISPR in clinical and virulent enterococci, but few have addressed the prevalence of these systems in environmental and commensal strains [16,17,18,19]. Additionally, CRISPR content in *E*. *faecalis* and *E*. *faecium* has been extensively reported, but a comprehensive survey including other *Enterococcus* species is lacking [15,17,18,20,21]. Since antiviral protection by CRISPR-Cas also prevents incorporation of potentially beneficial genes, retention of a CRISPR locus represents a tradeoff between protection and adaptability. To test the hypothesis that different habitats affect this tradeoff and thus the prevalence of CRISPR, our objective was to determine the frequency of active Type II CRISPR1 systems in *Enterococcus* species. Environmental, non-clinical enterococci were screened for presence of the conserved CRISPR1-*cas1* gene, as a marker for the active CRISPR locus most commonly detected in this genus. CRISPR1-*cas1* was detected in multiple *Enterococcus* species, including several not previously characterized as containing CRISPR systems. Significant differences in *cas1* incidence between species were also observed.

## Methods

### *Enterococcus* strains

*Enterococcus* isolates were cultured from activated sludge, oxygenated wastewater from residential and industrial sources, including storm runoff. Other samples included soil and sediment, compost, vegetation, marine and freshwater sources, and canine, feline, and avian fecal specimens (S1 Table). No permits were required for the described study, which complied with all relevant regulations. Water, soil, sediment, plant clippings, and fecal samples were taken from public properties where permission was not required, or from private property with permission of the owners. Activated sludge samples were supplied by water treatment plant supervisory personnel.

Activated sludge was diluted to 1:1000, and 10 mL of the dilution was filtered through 0.22-μm pore-size membrane filters, then incubated on mEnteroccocus agar (Difco) at 35°C for 24 hours. Isolated colonies were selected from the agar, and streaked for isolation of pure cultures on Enterococcosel agar (BBL). Environmental and fecal samples were enriched by incubation in azide dextrose broth for 24 hours at 35°C, followed by isolation of pure cultures on Enterococcosel agar. Additional *Enterococcus* strains from beach sand were isolated as previously described [22].

*Enterococcus faecalis* OG1RF (ATCC 47077), which contains a CRISPR1 locus, was selected as a positive control [20]. The strain was purchased from the American Type Culture Collection (Manassas, VA).

All isolates were Gram-positive, catalase-negative cocci. Species identity of all isolates was determined by 16S rRNA sequence match in the Ribosomal Database Project (http://rdp.cme.msu.edu/index.jsp), and identities were verified by 16S rRNA phylogenetic analysis. Isolate *cas1* sequences were confirmed to be *Enterococcus cas1* genes by BLASTn (NCBI) sequence match against the nucleotide collection (nr/nt) database.

### Identification of CRISPR components in available genome sequences

*Enterococcus cas1* genes for primer design were identified by BLASTn of the NCBI nucleotide collection (nr/nt) database, using the *E*. *faecalis* OG1RF *cas1* sequence (accession number CP002621.1) as the query (Fig A in S1 Text). CRISPR repeat-spacer arrays, and *cas* genes in proximity to the arrays, were investigated in 13 available *Enterococcus* genomes in CRISPRdb (*E*. *casseliflavus* EC20, accession number CP004856.1; *E*. *faecalis* 62, CP002491.1; *E*. *faecalis* D32, CP003726.1; *E*. *faecalis* OG1RF, CP002621.1; *E*. *faecalis* str. Symbioflor 1, HF558530.1; *E*. *faecalis* V583, AE016830.1; *E*. *faecium* Aus0004, CP003351.1; *E*. *faecium* Aus0085, CP006620.1; *E*. *faecium* DO, CP003583.1; *E*. *faecium* NRRL B-2354, CP004063.1; *E*. *hirae* ATCC 9790, CP003504.1; *E*. *mundtii* QU 25, AP013036.1; *Enterococcus* sp. 7L76, FP929058.1 [5]. Additional draft genomes (*E*. *durans* ATCC 6056, accession number GCA_000406985.1; *E*. *faecium* FB129-CNAB-4, GCA_000315405.1; *E*. *durans* IPLA 655, GCA_000350465.1) were downloaded from GenBank and analyzed for CRISPR content using CRISPRfinder (Table A in S1 Text) [6].

### PCR and sequencing

Nucleic acid extractions were performed using the MoBio UltraClean^®^ Microbial DNA Isolation Kit. The variable region of the 16S rRNA gene was amplified using universal bacterial DNA primers, forward, 5'-CCTACGGGAGGCAGCAG-3'; reverse, 5'-ATTACCGCGGCTGCTGG-3' [23].

To screen isolates for CRISPR1-*cas1*, primers amplifying a 212-bp internal region of the *cas1* gene (forward, 5’-ATGGGCTGGCGAAC-3’; reverse, 5’- CGCTTRTCATCGCAA-3’) were used. Multiple alignment of *Enterococcus* CRISPR1-*cas1* nucleotide sequences available at that time (*E*. *faecalis* OG1RF, accession number CP002621.1; *E*. *faecalis* D32, CP003726.1; *E*. *hirae* ATCC 9790, CP003504.1) was performed by MUSCLE [24,25] to locate conserved regions of the *cas1* homologs. Primers were designed manually, and their compatibility was confirmed using Primer3 (http://bioinfo.ut.ee/primer3/) [26,27]. Primers were deemed compatible, as Tm differed by 0.75°C, and no complementarity (self, pair, and primer hairpin) was detected. Target specificity of the primer set was further confirmed by Primer BLAST (http://www.ncbi.nlm.nih.gov/tools/primer-blast/) against all *Enterococcus* (taxid: 1350), using all variations of the reverse primer, which contains a degenerate base. The primer set amplified *in silico* in *E*. *faecalis* OG1RF, *E*. *faecalis* D32, and *E*. *hirae* ATCC 9790. Amplification was optimized for the following program: 2 minutes at 94°C, 30 cycles of [1 minute at 94°C, 1 minute at 48.9°C, 1 minute at 72°C], 10 minutes at 72°C.

PCR products were submitted to Massachusetts General Hospital DNA Sequencing Core Facility or Eton Biosciences, Boston, MA for sequencing. Sequences were curated manually, and 16S rRNA gene sequences were deposited in GenBank (S1 Table).

### Analysis and phylogeny

To test whether CRISPR1-*cas1* distribution significantly differed by species or source, data were analyzed by Chi square and Fisher’s exact tests (Tables 1–3).

**Table 1:** Detection of CRISPR1-*cas1* in all *Enterococcus* strains, by source of isolate. Differences between sources are not significant, P value = 0.6598.

Source	*cas1*-positive	No. of isolates	% cas1- positives
Activated sludge	40	131	30.5
Environmental samples	38	113	33.6
Animal fecal	12	31	38.7

**Table 2:** Detection of CRISPR1-*cas1* in *E*. *faecalis* strains, by source of isolate. Differences between sources are not significant, P value = 0.6166.

Source	*cas1*-positive	No. of isolates	% cas1- positives
Activated sludge	9	38	23.7
Environmental samples	17	69	24.6
Animal fecal	0	3	-

**Table 3:** Detection of CRISPR1-*cas1* in *E*. *hirae* strains, by source of isolate. Differences between sources are not significant, P value = 0.3302.

Source	*cas1*-positive	No. of isolates	% cas1 positives
Activated sludge	29	39	74.4
Environmental samples	16	25	64.0
Animal fecal	12	14	85.7

Phylogeny was constructed using SeaView 4 (http://doua.prabi.fr/software/seaview). Multiple sequence alignment was performed within Seaview 4 using MUSCLE [25], and gap-only sites were removed. A maximum likelihood tree (PhyML) was generated, using the GTR model and aLRT branch support, with all other parameters set to default (nucleotide equilibrium frequencies: empirical; Ts/Tv ratio: fixed, 4.0; invariable sites: none; across site rate variation: optimized; tree searching operations: NNI; starting tree: BioNJ, optimized tree topology). FigTree 1.4.2 (http://tree.bio.ed.ac.uk/software/figtree) was used for tree visualization. Two of the sequences used to design the *cas1* primers were used as reference sequences in the *cas1* phylogenetic tree; *E*. *faecalis* D32 was omitted, as it is identical to that of *E*. *faecalis* OG1RF.

## Results

The predominant *Enterococcus* species isolated were *E*. *faecalis* (40.0% of 275 total isolates), *E*. *hirae* (28.4%), *E*. *durans* (20.4%), and *E*. *faecium* (5.1%). Additional enterococcal species were isolated less frequently, and include *E*. *casseliflavus*, *E*. *sulfureus*, *E*. *mundtii*, *E*. *malodoratus*, *E*. *termitis*, and *E*. *sanguinicola* (Table 4).

**Table 4:** Detection of CRISPR1-*cas1* in *Enterococcus*, by species. Differences in CRISPR1-*cas1* detection between *E*. *faecalis*, *E*. *hirae*, and *E*. *durans* isolates are significant, P value < 0.0001. Species for which a low number of strains were isolated are indicated in italics.

Species	Cas1-positives	Total isolates	Percent cas1 positive
*E*. *faecalis*	26	110	23.6
*E*. *hirae*	57	78	73.1
*E*. *durans*	4	56	7.1
*E*. *faecium*	*1*	*14*	*7*.*1*
*E*. *casseliflavus*	*1*	*7*	*14*.*3*
*E*. *sulfureus*	*1*	*2*	*50*.*0*
*E*. *mundtii*	*0*	*2*	*0*.*0*
*E*. *sanguinicola*	*0*	*1*	*0*.*0*
*E*. *malodoratus*	*0*	*4*	*0*.*0*
*E*. *termitis*	*0*	*1*	*0*.*0*
**Total**	90	275	32.7

**Fig 1:**
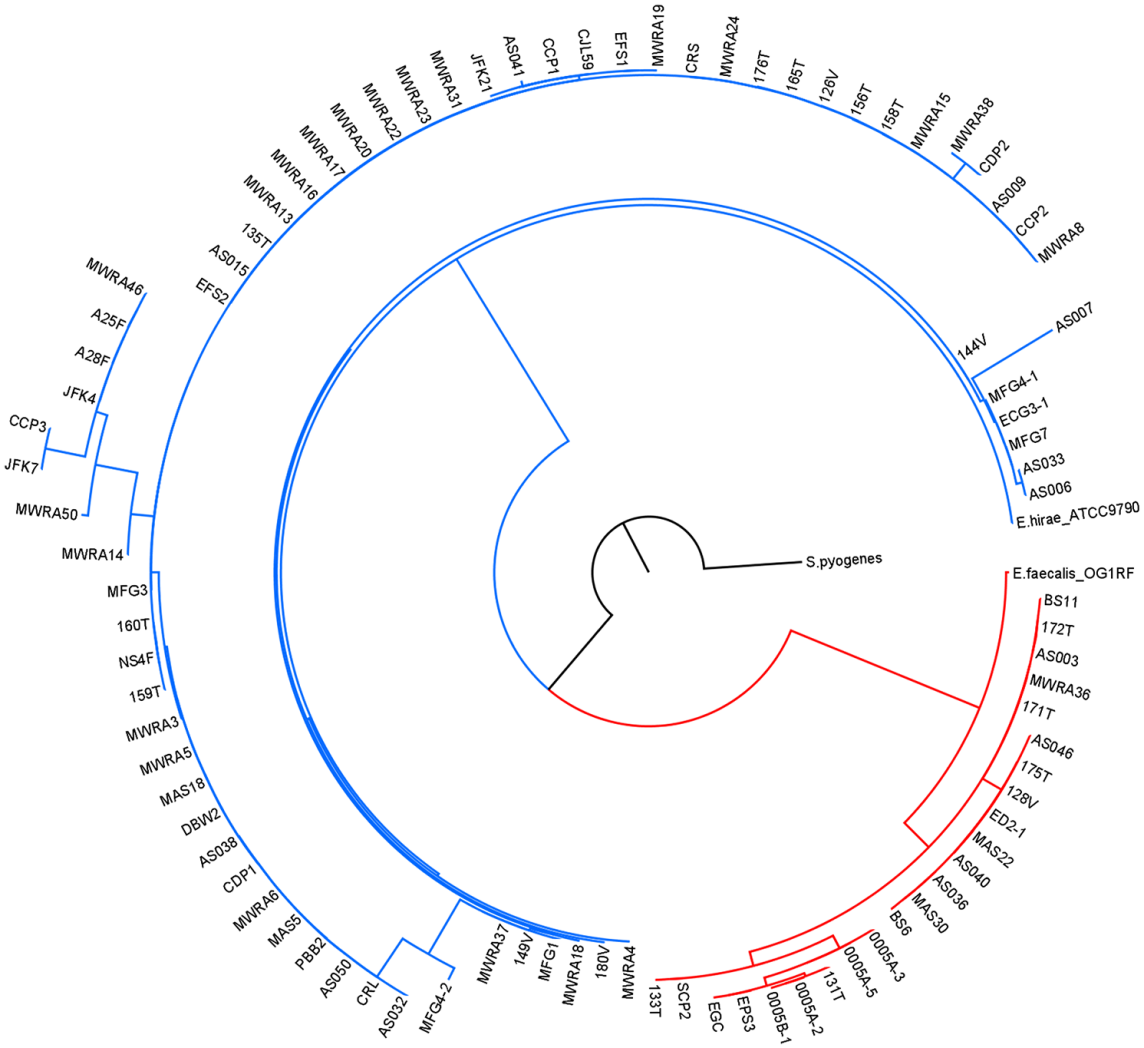
Phylogenetic tree of CRISPR1-*cas1* partial sequences. Red branches represent the *E*. *faecalis*-like *cas1* cluster; blue branches represent the *E*. *hirae cas1* cluster.

The CRISPR1-*cas1* gene was detected in 32.7% of all *Enterococcus* isolates (Table 4). Within the three most predominant species isolated, frequency of *cas1* detection varied significantly. The incidence of CRISPR1-*cas1* genes between *E*. *faecalis*, *E*. *durans*, and *E*. *hirae* is significantly different (Table 4; p < 0.0001). The frequency of remaining species was not considered in this analysis due to small sample size. CRISPR1-*cas1* was detected in 23.6% of *E*. *faecalis* isolates, while 73.1% of *E*. *hirae* and 7.1% of *E*. *durans* strains contain the gene. *Cas1* was also detected in isolates of *E*. *faecium*, *E*. *casseliflavus* and *E*. *sulfureus*. The few strains of *E*. *malodoratus*, *E*. *sanguinicola*, *E*. *mundtii*, and *E*. *termitis* that were isolated did not contain *cas1* (Table 4). The origin of the bacterial strain and presence of a CRISPR1-*cas1* gene were not significantly correlated. This observation was consistent for all *Enterococcus* species analyzed, as well as intraspecific analyses of the two most commonly isolated species, *E*. *faecalis* and *E*. *hirae* (Tables 1–3). A phylogenetic tree of partial *cas1* sequences formed two strongly distinct clusters around the *E*. *faecalis* OG1RF and the *E*. *hirae* ATCC 9790 *cas1* reference sequences (Fig 1). All but 4 of the 26 *E*. *faecalis cas1* genes clustered with the *E*. *faecalis* OG1RF-like *cas1* gene. The remaining four strains of *E*. *faecalis* (MWRA37, MWRA22, 176T, and 158T) contained an *E*. *hirae*-like *cas1* homolog. All identified *E*. *hirae* strains possess a *cas1* homolog similar to that of *E*. *hirae* ATCC 9790. *Cas1* sequences for *E*. *casseliflavus*, *E*. *faecium*, and *E*. *sulfureus* share identity with the *E*. *hirae* gene. *E*. *durans* strains contained *cas1* genes homologous to both the *E*. *faecalis* OG1RF and *E*. *hirae* ATCC9790 *cas1* types.

**Fig 2:**
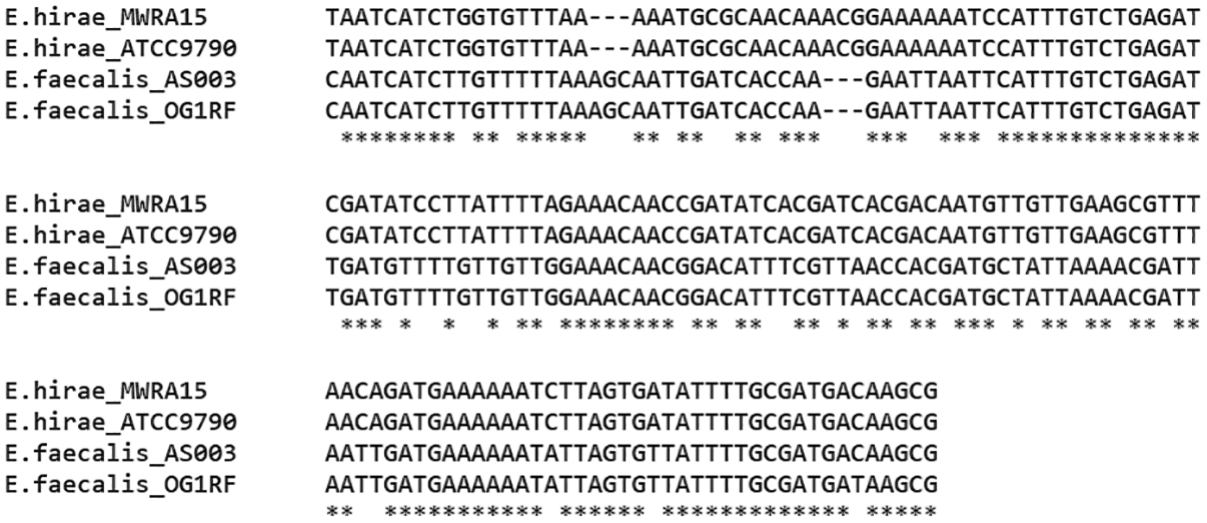
Comparison of partial CRISPR1-*cas1* sequences. Representative isolates (*E*. *hirae* MWRA15 and *E*. *faecalis* AS003) and reference strains (*E*. *hirae* ATCC 9790 and *E*. *faecalis* OG1RF) were aligned using MUSCLE. Bases conserved between all analyzed sequences are indicated with asterisks; spaces denote transitions and transversions, and dashes represent indel regions.

*Cas1* sequences are conserved in the region amplified in this study, and the *E*. *hirae* and *E*. *faecalis* homologs are distinctly different from each other, perhaps reflecting species-level evolution. Within this region, the sequences differ by 16 transitions, 20 transversions, and a 3 bp indel, and not a continuum of differences between the two clusters (Fig 2). *E*. *faecalis* strains usually contain an *E*. *faecalis cas1* homolog, and *E*. *hirae*-like *cas1* genes typically appear in strains identified as *E*. *hirae*. Additionally, three of four *E*. *durans cas1*-positive strains contain *E*. *hirae* homologs, but one contains an *E*. *faecalis*-like gene. Horizontal transfer of CRISPR components in enterococci has yet to be demonstrated.

## Discussion

### Incidence of *cas1* in *Enterococcus*

This study is the first systematic analysis of Type II CRISPR1-Cas incidence in non-clinical enterococci. The incidence of CRISPR1-associated *cas1* in *E*. *hirae* (73.1%) is significantly higher than in *E*. *faecalis* (Table 4). *E*. *faecalis* is a human commensal species, and selective pressure for antibiotic resistance may be high [15]. If the selective pressure for adapting to antibiotics in the human gut environment is higher than the selective pressure by lytic bacteriophage, then lower incidence of CRISPR-Cas is expected for species in habitats with higher phage pressure. The phage pressure in the typical habitats of *E*. *hirae* are not characterized, but *E*. *hirae* is primarily associated with animals, including birds, household pets, and livestock. Although *E*. *hirae* is implicated in animal disease, it is very rarely pathogenic to humans [28]. As CRISPR presence is inversely correlated with acquisition of traits such as antibiotic resistance in enterococci, widespread distribution of CRISPR1-*cas1* within this species may correspond with its lack of virulence.

An effect of the source of isolated enterococci was not observed; however, activated sludge contains wastewater influent from a variety of sources, including household, commercial, and clinical sewers, as well as storm drain runoff. Therefore, differentiating bacterial isolates by host species origin from a common source is problematic. Additionally, environmental samples, such as the beach sand and sediment used in this study, may be influenced by human or animal presence, and should not be considered autochthonous [29]. Thus, it is not possible to conclusively compare strain origin or source and CRISPR-Cas presence in the current study. This remains an area for future research.

### Cas1 phylogeny indicates horizontal transfer of CRISPR loci

The tight clustering of the partial *cas1* sequence phylogeny was striking. Therefore, the four strains of *E*. *faecalis* that clustered with the *E*. *hirae cas1* sequences indicates horizontal transfer of CRISPR elements between *Enterococcus* species (Fig 1). CRISPR1-*cas1* genes identified in *E*. *sulfureus*, *E*. *casseliflavus*, and *E*. *faecium* cluster with the *cas1* homologs in *E*. *hirae* strains (Fig 1). This is further indication of horizontal transfer, or CRISPR-Cas systems may be conserved with high levels of sequence similarity between these species. A more comprehensive description of the CRISPR-Cas systems in *E*. *durans*, *E*. *faecium*, *E*. *casseliflavus*, and *E*. *sulfureus* is needed to answer this question, as well as to shed light on differences in *cas* genes and array content that may explain interspecific CRISPR diversity.

### CRISPR1 in *E*. *durans*, *E*. *casseliflavus*, and *E*. *sulfureus*

This is the first report of the presence of CRISPR1-Cas systems in *E*. *durans*, *E*. *casseliflavus*, and *E*. *sulfureus*. *E*. *durans* is a minor component of human and animal gut flora, and is also found in food of animal origin, especially dairy products [11,28]. Lack of virulence genes, including those that confer antibiotic resistance, indicate a probiotic role for *E*. *durans* [30]. CRISPR1 incidence in *E*. *durans* is low, but phage pressure in typical habitats of this species are not well characterized. *E*. *casseliflavus* and *E*. *sulfureus* are primarily plant-associated species [31,32,33]. Recent studies of *E*. *casseliflavus* have implicated the bacterium in human infection; however, these cases remain infrequent [34,35,36,37]. Reports implicating *E*. *sulfureus* in human disease could not be found in scientific literature. The rarity with which these species are pathogenic suggests an inverse correlation between virulence and CRISPR content, as was demonstrated in *Escherichia coli* [15,38,39]. Accurate frequencies of CRISPR1 loci in these species will require more comprehensive testing. In this study, only a few isolates of these species were cultured and screened for the *cas1* gene.

CRISPR1-*cas1* was not detected in isolates of *E*. *mundtii* and *E*. *malodoratus*. CRISPR loci have not been reported in two *E*. *mundtii* genomes previously analyzed, and incidence in *E*. *malodoratus* has also not been reported [40,41]. However, these sample sizes are too small to conclude that these species do not possess CRISPR1 loci. Additionally, the Type II-specific *cas1* primers used in this study are unlikely to amplify all *cas1* genes within *Enterococcus*, as species may contain CRISPR-Cas systems of different types [42]. With the three additional species reported here to contain CRISPR1-*cas1*, six species of *Enterococcus* are reported to possess CRISPR. But, as many as 40 other *Enterococcus* species have yet to be investigated [43]. Although more thorough characterization is warranted, the presence of *cas* genes in the species reported here indicates that CRISPR1-Cas systems may be widespread among the *Enterococcus* genus. The primers designed here successfully amplified a conserved region of the *cas1* gene in multiple enterococcal species, making it an efficient marker for screening for CRISPR1 loci. Furthermore, widespread incidence of active CRISPRs and omnipresence of the clade in many environments make *Enterococcus* an ideal model for investigation of CRISPR dynamics.

### Conclusions

Immunity against lytic phages is a recognizable evolutionary benefit for a bacterium, demonstrated both experimentally and in mathematical models of CRISPR-Cas/phage interaction [1,44]. Often considered as beneficial, indiscriminate insertion of foreign genetic elements, such as genomic islands, prophages and plasmids, on the other hand, can result in disruption of essential gene function and incorrect regulation of acquired genes [45]. CRISPR-mediated prevention of these detrimental insertions may also confer an evolutionary advantage [2]. However, horizontally-acquired genes may increase fitness by conferring habitat adaptations. Such adaptations in *Enterococcus* include antibiotic resistance, enhanced biofilm formation, resistance to metal toxicity, and expanded metabolic capacity [46,47]. Maintaining a functional CRISPR-Cas system also incurs an energetic cost for the organism [45]. Thus, for the bacterium possessing CRISPR loci, there is a tradeoff between adaptability and protection. Significant differences in Type II CRISPR1-*cas1* incidence seen here indicate that selective pressures exerted by this tradeoff may influence CRISPR-Cas distribution in a species-dependent manner. The nature of this selection remains an area of future research.

## Supporting Information

S1 Fig Phylogenetic tree of *Enterococcus* isolates. (TIF)

S1 Table 16S rRNA gene sequences and accession numbers deposited in GenBank. (DOCX)

S1 Text Supplemental Methods. (DOCX)
