# Supplementary figures and images for: Incidence of Type II CRISPR1-Cas Systems in Enterococcus Is Species-Dependent
Source: PLoS One. 2015 Nov 24;10(11):e0143544. doi: 10.1371/journal.pone.0143544 (PMC4658022; doi:10.1371/journal.pone.0143544)

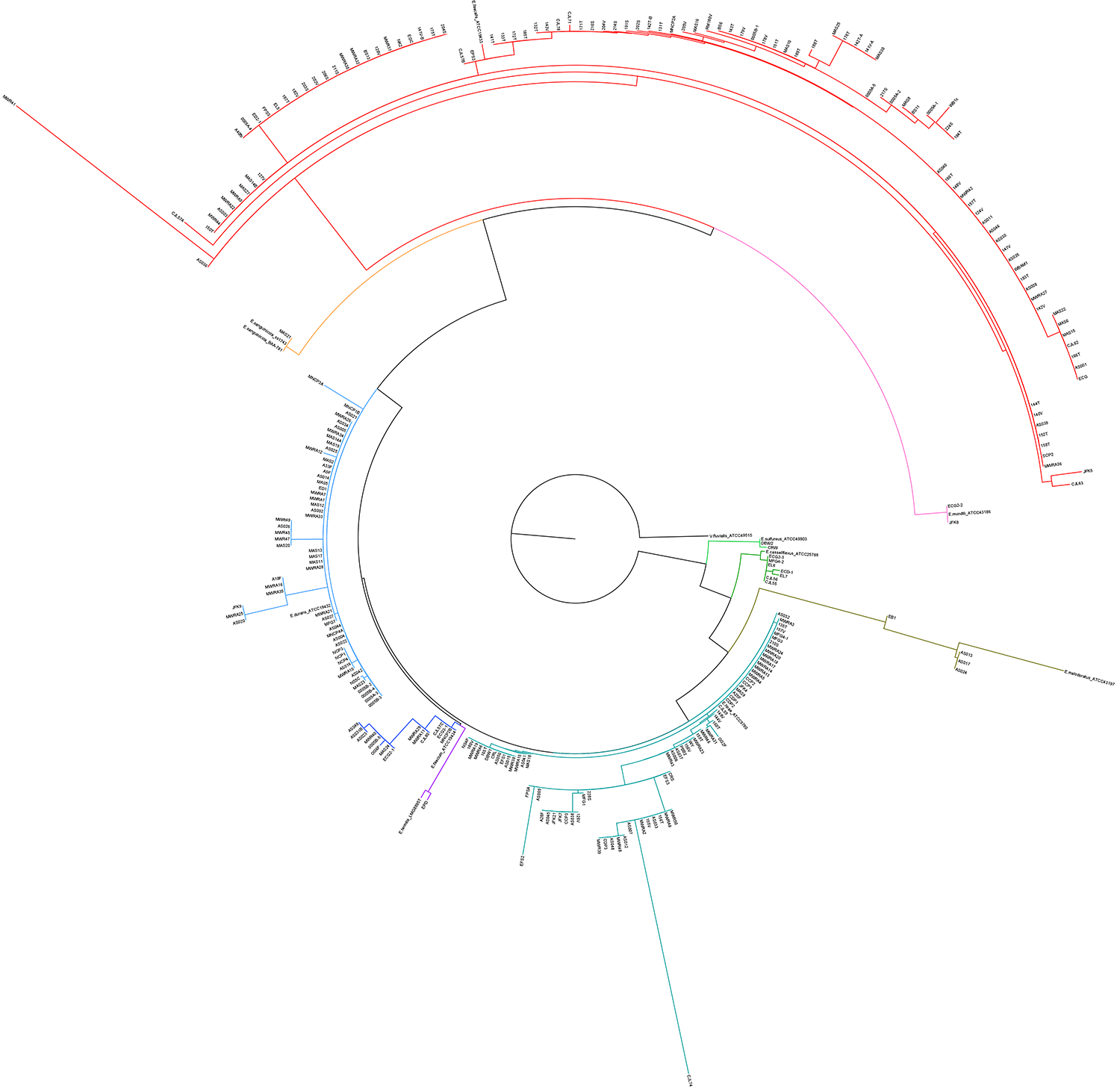

Supplement: S1 Fig — (TIF) [file pone.0143544.s001.tif]
