# Supplementary material for: Incidence of Type II CRISPR1-Cas Systems in Enterococcus Is Species-Dependent
Source: PLoS One. 2015 Nov 24;10(11):e0143544. doi: 10.1371/journal.pone.0143544 (PMC4658022; doi:10.1371/journal.pone.0143544)
Supplement: S1 Table — (DOCX) [file pone.0143544.s002.docx]

Table S1: Enterococcus strains in this study.

| Strain ID | Species | Sample Source | Sample Site | *cas1* | GenBank Accession Number |
| --- | --- | --- | --- | --- | --- |
| 0005A-1 | *E. faecalis* | Sand | Wollaston Beach, Quincy, MA | N | KT205732 |
| 0005A-2 | *E. faecalis* | Sand | Wollaston Beach, Quincy, MA | P | KT205733 |
| 0005A-3 | *E. durans* | Sand | Wollaston Beach, Quincy, MA | P | KT205734 |
| 0005A-4 | *E. faecalis* | Sand | Wollaston Beach, Quincy, MA | N | KT205735 |
| 0005A-5 | *E. faecalis* | Sand | Wollaston Beach, Quincy, MA | P | KT205736 |
| 0005B-1 | *E. faecalis* | Sand | Wollaston Beach, Quincy, MA | P | KT205737 |
| 0005B-2 | *E. durans* | Sand | Wollaston Beach, Quincy, MA | N | KT205738 |
| 0005B-3 | *E. durans* | Sand | Wollaston Beach, Quincy, MA | N | KT205739 |
| 0005B-4 | *E. durans* | Sand | Wollaston Beach, Quincy, MA | N | KT205740 |
| 0005B-5 | *E. faecium* | Sand | Wollaston Beach, Quincy, MA | N | KT205741 |
| 124V | *E. faecalis* | *Spartina alterniflora* | Blacks Creek, Quincy, MA | N | KT205687 |
| 126V | *E. hirae* | *Spartina alterniflora* | Blacks Creek, Quincy, MA | P | KT205688 |
| 127V | *E. faecalis* | *Spartina alterniflora* | Blacks Creek, Quincy, MA | N | KT205689 |
| 128V | *E. faecalis* | *Spartina alterniflora* | Blacks Creek, Quincy, MA | P | KT205690 |
| 131T | *E. faecalis* | Topsoil | Blacks Creek, Quincy, MA | P | KT205691 |
| 132T | *E. faecalis* | Topsoil | Blacks Creek, Quincy, MA | N | KT205692 |
| 133T | *E. faecalis* | Topsoil | Blacks Creek, Quincy, MA | P | KT205693 |
| 135T | *E. hirae* | Topsoil | Blacks Creek, Quincy, MA | P | KT205694 |
| 140V | *E. faecalis* | *Spartina alterniflora* | Blacks Creek, Quincy, MA | N | KT205695 |
| 141T | *E. faecalis* | Topsoil | Blacks Creek, Quincy, MA | N | KT205696 |
| 141V-A | *E. faecalis* | *Spartina alterniflora* | Blacks Creek, Quincy, MA | N | KT205697 |
| 141V-B | *E. faecalis* | *Spartina alterniflora* | Blacks Creek, Quincy, MA | N | KT205698 |
| 142T-A | *E. faecalis* | Topsoil | Blacks Creek, Quincy, MA | N | KT205699 |
| 142T-B | *E. faecalis* | Topsoil | Blacks Creek, Quincy, MA | N | KT205700 |
| 142V | *E. faecalis* | *Spartina alterniflora* | Blacks Creek, Quincy, MA | N | KT205701 |
| 143T | *E. faecalis* | Topsoil | Blacks Creek, Quincy, MA | N | KT205702 |
| 143V | *E. faecalis* | *Spartina alterniflora* | Blacks Creek, Quincy, MA | N | KT205703 |
| 144T | *E. faecalis* | Topsoil | Blacks Creek, Quincy, MA | N | KT205704 |
| 144V | *E. hirae* | *Spartina alterniflora* | Blacks Creek, Quincy, MA | P | KT205705 |
| 147V | *E. faecalis* | *Spartina alterniflora* | Blacks Creek, Quincy, MA | N | KT205706 |
| 148V | *E. faecalis* | *Spartina alterniflora* | Blacks Creek, Quincy, MA | N | KT205707 |
| 149V | *E. hirae* | *Spartina alterniflora* | Blacks Creek, Quincy, MA | P | KT205708 |
| 150T | *E. faecalis* | Topsoil | Blacks Creek, Quincy, MA | N | KT205709 |
| 151T | *E. faecalis* | Topsoil | Blacks Creek, Quincy, MA | N | KT205710 |
| 152T | *E. faecalis* | Topsoil | Blacks Creek, Quincy, MA | N | KT205711 |
| 155V | *E. hirae* | *Spartina alterniflora* | Blacks Creek, Quincy, MA | N | KT205712 |
| 156T | *E. hirae* | Topsoil | Blacks Creek, Quincy, MA | P | KT205713 |
| 156V | *E. hirae* | *Spartina alterniflora* | Blacks Creek, Quincy, MA | N | KT205714 |
| 157T | *E. faecalis* | Topsoil | Blacks Creek, Quincy, MA | N | KT205715 |
| 157V | *E. hirae* | *Spartina alterniflora* | Blacks Creek, Quincy, MA | N | KT205716 |
| 158T | *E. faecalis* | Topsoil | Blacks Creek, Quincy, MA | N | KT254126 |
| 159T | *E. hirae* | Topsoil | Blacks Creek, Quincy, MA | P | KT205717 |
| 160T | *E. hirae* | Topsoil | Blacks Creek, Quincy, MA | P | KT205718 |
| 165T | *E. hirae* | Topsoil | Blacks Creek, Quincy, MA | P | KT205719 |
| 166T | *E. faecalis* | Topsoil | Blacks Creek, Quincy, MA | N | KT205572 |
| 171T | *E. faecalis* | Topsoil | Blacks Creek, Quincy, MA | P | KT205723 |
| 172T | *E. faecalis* | Topsoil | Blacks Creek, Quincy, MA | P | KT205720 |
| 175T | *E. faecalis* | Topsoil | Blacks Creek, Quincy, MA | P | KT205724 |
| 176T | *E. faecalis* | Topsoil | Blacks Creek, Quincy, MA | P | KT205725 |
| 178V | *E. faecalis* | *Spartina alterniflora* | Rowley Marsh, Rowley, MA | N | KT205742 |
| 179V | *E. faecalis* | *Spartina alterniflora* | Rowley Marsh, Rowley, MA | N | KT205743 |
| 180V | *E. hirae* | *Spartina alterniflora* | Blacks Creek, Quincy, MA | P | KT205726 |
| 182T | *E. faecalis* | Topsoil | Blacks Creek, Quincy, MA | N | KT254127 |
| 182V | *E. faecalis* | *Spartina alterniflora* | Rowley Marsh, Rowley, MA | N | KT205745 |
| 183T | *E. faecalis* | Topsoil | Blacks Creek, Quincy, MA | N | KT205727 |
| 184T | *E. faecalis* | Topsoil | Blacks Creek, Quincy, MA | N | KT205728 |
| 185T | *E. faecalis* | Topsoil | Blacks Creek, Quincy, MA | N | KT205721 |
| 186T | *E. faecalis* | Topsoil | Blacks Creek, Quincy, MA | N | KT205722 |
| 187T | *E. faecalis* | Topsoil | Blacks Creek, Quincy, MA | N | KT205729 |
| 188T | *E. faecalis* | Topsoil | Blacks Creek, Quincy, MA | N | KT205573 |
| 189T | *E. faecalis* | Topsoil | Blacks Creek, Quincy, MA | N | KT205574 |
| 191S | *E. faecalis* | Sediment | Rowley Marsh, Rowley, MA | N | KT205749 |
| 202S | *E. faecalis* | Sediment | Rowley Marsh, Rowley, MA | N | KT205750 |
| 202V | *E. faecalis* | *Spartina alterniflora* | Rowley Marsh, Rowley, MA | N | KT205746 |
| 203S | *E. faecalis* | Sediment | Rowley Marsh, Rowley, MA | N | KT205751 |
| 204S | *E. faecalis* | Sediment | Rowley Marsh, Rowley, MA | N | KT205752 |
| 204V | *E. faecalis* | *Spartina alterniflora* | Rowley Marsh, Rowley, MA | N | KT205747 |
| 205V | *E. faecalis* | *Spartina alterniflora* | Rowley Marsh, Rowley, MA | N | KT205748 |
| 206S | *E. faecalis* | Sediment | Rowley Marsh, Rowley, MA | N | KT205753 |
| 210S | *E. hirae* | Sediment | Rowley Marsh, Rowley, MA | N | KT205754 |
| 211S | *E. faecalis* | Sediment | Rowley Marsh, Rowley, MA | N | KT205755 |
| 214S | *E. faecalis* | Sediment | Rowley Marsh, Rowley, MA | N | KT205756 |
| 216S | *E. faecalis* | Sediment | Rowley Marsh, Rowley, MA | N | KT205757 |
| 217S | *E. faecalis* | Sediment | Rowley Marsh, Rowley, MA | N | KT205758 |
| 224S | *E. faecalis* | Sediment | Rowley Marsh, Rowley, MA | N | KT205759 |
| 228S | *E. hirae* | Sediment | Rowley Marsh, Rowley, MA | N | KT205760 |
| A10F | *E. durans* | Pigeon feces | Airport MBTA Station | N | KT205577 |
| A25F | *E. hirae* | Pigeon feces | Airport MBTA Station | P | KT205616 |
| A28F | *E. hirae* | Pigeon feces | Airport MBTA Station | P | KT205578 |
| A33F | *E. durans* | Pigeon feces | Airport MBTA Station | N | KT205579 |
| A40fb | *E. faecalis* | Pigeon feces | Airport MBTA Station | N | KT205731 |
| A5F | *E. durans* | Pigeon feces | Airport MBTA Station | N | KT205615 |
| AS001 | *E. faecalis* | Activated sludge | Deer Island W.T. Plant | N | KT205773 |
| AS002 | *E. durans* | Activated sludge | Deer Island W.T. Plant | N | KT205774 |
| AS003 | *E. faecalis* | Activated sludge | Deer Island W.T. Plant | P | KT205775 |
| AS004 | *E. durans* | Activated sludge | Deer Island W.T. Plant | N | KT205776 |
| AS005 | *E. durans* | Activated sludge | Deer Island W.T. Plant | N | KT205777 |
| AS006 | *E. hirae* | Activated sludge | Deer Island W.T. Plant | P | KT205778 |
| AS007 | *E. hirae* | Activated sludge | Deer Island W.T. Plant | P | KT205779 |
| AS008 | *E. faecalis* | Activated sludge | Deer Island W.T. Plant | N | KT205780 |
| AS009 | *E. hirae* | Activated sludge | Deer Island W.T. Plant | P | KT205781 |
| AS011 | *E. faecalis* | Activated sludge | Deer Island W.T. Plant | N | KT205782 |
| AS012 | *E. hirae* | Activated sludge | Deer Island W.T. Plant | N | KT205783 |
| AS013 | *E. malodoratus* | Activated sludge | Deer Island W.T. Plant | N | KT205784 |
| AS014 | *E. durans* | Activated sludge | Deer Island W.T. Plant | N | KT205785 |
| AS015 | *E. hirae* | Activated sludge | Deer Island W.T. Plant | P | KT205786 |
| AS016 | *E. durans* | Activated sludge | Deer Island W.T. Plant | N | KT205787 |
| AS017 | *E. malodoratus* | Activated sludge | Deer Island W.T. Plant | N | KT205788 |
| AS020 | *E. durans* | Activated sludge | Deer Island W.T. Plant | N | KT205789 |
| AS021 | *E. durans* | Activated sludge | Deer Island W.T. Plant | N | KT205790 |
| AS022 | *E. durans* | Activated sludge | Deer Island W.T. Plant | N | KT205791 |
| AS023 | *E. faecium* | Activated sludge | Deer Island W.T. Plant | N | KT205792 |
| AS024 | *E. malodoratus* | Activated sludge | Deer Island W.T. Plant | N | KT205793 |
| AS025 | *E. durans* | Activated sludge | Deer Island W.T. Plant | N | KT205794 |
| AS026 | *E. durans* | Activated sludge | Deer Island W.T. Plant | N | KT205795 |
| AS027 | *E. durans* | Activated sludge | Deer Island W.T. Plant | N | KT205796 |
| AS030 | *E. faecalis* | Activated sludge | Deer Island W.T. Plant | N | KT205797 |
| AS031B | *E. faecium* | Activated sludge | Deer Island W.T. Plant | N | KT205798 |
| AS032 | *E. hirae* | Activated sludge | Deer Island W.T. Plant | P | KT205799 |
| AS033 | *E. hirae* | Activated sludge | Deer Island W.T. Plant | P | KT205800 |
| AS034 | *E. durans* | Activated sludge | Deer Island W.T. Plant | N | KT205801 |
| AS035 | *E. faecalis* | Activated sludge | Deer Island W.T. Plant | N | KT205802 |
| AS036 | *E. faecalis* | Activated sludge | Deer Island W.T. Plant | P | KT205803 |
| AS037 | *E. hirae* | Activated sludge | Deer Island W.T. Plant | N | KT205804 |
| AS038 | *E. hirae* | Activated sludge | Deer Island W.T. Plant | P | KT205805 |
| AS039 | *E. faecalis* | Activated sludge | Deer Island W.T. Plant | N | KT205806 |
| AS040 | *E. faecalis* | Activated sludge | Deer Island W.T. Plant | P | KT205807 |
| AS041 | *E. hirae* | Activated sludge | Deer Island W.T. Plant | P | KT205808 |
| AS042 | *E. durans* | Activated sludge | Deer Island W.T. Plant | N | KT205809 |
| AS044 | *E. durans* | Activated sludge | Deer Island W.T. Plant | N | KT205810 |
| AS045 | *E. hirae* | Activated sludge | Deer Island W.T. Plant | N | KT205811 |
| AS046 | *E. faecalis* | Activated sludge | Deer Island W.T. Plant | P | KT205812 |
| AS048 | *E. hirae* | Activated sludge | Deer Island W.T. Plant | N | KT205813 |
| AS049 | *E. faecium* | Activated sludge | Deer Island W.T. Plant | N | KT205814 |
| AS050 | *E. hirae* | Activated sludge | Deer Island W.T. Plant | P | KT205815 |
| BS11 | *E. faecalis* | Sand | Carson Beach | P | KT205833 |
| BS13 | *E. faecalis* | Sand | Castle Island Beach | N | KT205834 |
| BS6 | *E. faecalis* | Sand | King's Beach Outfall | P | KT205835 |
| CCP1 | *E. hirae* | Feline feces | Winthrop, MA | P | KT205630 |
| CCP2 | *E. hirae* | Feline feces | Winthrop, MA | P | KT205631 |
| CCP3 | *E. hirae* | Feline feces | Winthrop, MA | P | KT205632 |
| CDP1 | *E. hirae* | Canine feces | Winthrop, MA | P | KT205627 |
| CDP2 | *E. hirae* | Canine feces | Winthrop, MA | P | KT205628 |
| CDP3 | *E. hirae* | Canine feces | Winthrop, MA | N | KT205629 |
| CJL55 | *E. casseliflavus* | Activated sludge | Deer Island W.T. Plant | N | KT205639 |
| CJL56 | *E. casseliflavus* | Activated sludge | Deer Island W.T. Plant | N | KT205640 |
| CJL57A | *E. faecalis* | Activated sludge | Deer Island W.T. Plant | N | KT205641 |
| CJL57B | *E. faecalis* | Activated sludge | Deer Island W.T. Plant | N | KT205642 |
| CJL57C | *E. faecium* | Activated sludge | Deer Island W.T. Plant | N | KT205643 |
| CJL59 | *E. hirae* | Activated sludge | Deer Island W.T. Plant | P | KT205644 |
| CJL62 | *E. faecalis* | Activated sludge | Deer Island W.T. Plant | N | KT205645 |
| CJL63 | *E. faecalis* | Activated sludge | Deer Island W.T. Plant | N | KT205646 |
| CJL71 | *E. faecalis* | Activated sludge | Deer Island W.T. Plant | N | KT205647 |
| CJL74 | *E. hirae* | Activated sludge | Deer Island W.T. Plant | N | KT205648 |
| CJL78 | *E. faecalis* | Activated sludge | Deer Island W.T. Plant | N | KT205649 |
| CJL80 | *E. faecium* | Activated sludge | Deer Island W.T. Plant | N | KT205650 |
| CRL | *E. hirae* | Water | Charles River | P | KT205769 |
| CRS | *E. hirae* | Water | Charles River | P | KT205770 |
| CRW | *E. sulfureus* | Water | Charles River | N | KT205768 |
| DBW1 | *E. hirae* | Water | Donovan Beach, Winthrop, MA | N | KT205766 |
| DBW2 | *E. sulfureus* | Water | Donovan Beach, Winthrop, MA | P | KT205767 |
| EB1 | *E. malodoratus* | Unknown environmental | Cherry Hill Park, Danvers | N | KT205832 |
| ECD-1 | *E. casseliflavus* | Compost | Marshfield, MA | N | KT205826 |
| ECG | *E. faecalis* | Compost | Marshfield, MA | N | KT205827 |
| ECG2-1 | *E. faecium* | Unknown environmental | Cherry Hill Park, Danvers | N | KT205828 |
| ECG2-2 | *E. mundtii* | Unknown environmental | Cherry Hill Park, Danvers | N | KT205829 |
| ECG2-3 | *E. casseliflavus* | Unknown environmental | Cherry Hill Park, Danvers | N | KT205830 |
| ECG3-1 | *E. faecium* | Unknown environmental | Cherry Hill Park, Danvers | P | KT254128 |
| ED1 | *E. durans* | Unknown environmental | Roslindale | N | KT205819 |
| ED2-1 | *E. faecalis* | Unknown environmental | Roslindale | P | KT205820 |
| EFS1 | *E. hirae* | Yard waste | Winthrop, MA | P | KT205821 |
| EFS2 | *E. hirae* | Yard waste | Winthrop, MA | P | KT205822 |
| EFS3 | *E. hirae* | Yard waste | Winthrop, MA | N | KT205823 |
| EGC | *E. faecalis* | Grass clippings | Marshfield, MA | P | KT205825 |
| EL5 | *E. faecalis* | Unknown environmental | Roslindale | N | KT205816 |
| EL6 | *E. casseliflavus* | Unknown environmental | Roslindale | N | KT205817 |
| EL7 | *E. casseliflavus* | Unknown environmental | Roslindale | N | KT205818 |
| EPD | *E. termitis* | Unknown environmental | Cherry Hill Park, Danvers | N | KT205831 |
| EPS3 | *E. faecalis* | Yard waste | Winthrop, MA | P | KT205824 |
| FPSA | *E. hirae* | Unknown environmental | Foster's Pond | N | KT205771 |
| FPSS | *E. faecalis* | Unknown environmental | Foster's Pond | N | KT205772 |
| JFK21 | *E. hirae* | Pigeon feces | JFK MBTA Station | P | KT205613 |
| JFK4 | *E. hirae* | Pigeon feces | JFK MBTA Station | P | KT205610 |
| JFK5 | *E. faecalis* | Pigeon feces | JFK MBTA Station | N | KT205730 |
| JFK7 | *E. hirae* | Pigeon feces | JFK MBTA Station | P | KT205611 |
| JFK8 | *E. mundtii* | Pigeon feces | JFK MBTA Station | N | KT205612 |
| JFK9 | *E. durans* | Pigeon feces | JFK MBTA Station | N | KT205575 |
| MAS10 | *E. faecalis* | Activated sludge | Deer Island W.T. Plant | N | KT205593 |
| MAS11 | *E. durans* | Activated sludge | Deer Island W.T. Plant | N | KT205594 |
| MAS12 | *E. durans* | Activated sludge | Deer Island W.T. Plant | N | KT205595 |
| MAS13 | *E. durans* | Activated sludge | Deer Island W.T. Plant | N | KT205596 |
| MAS14A | *E. durans* | Activated sludge | Deer Island W.T. Plant | N | KT205597 |
| MAS14B | *E. faecalis* | Activated sludge | Deer Island W.T. Plant | N | KT254129 |
| MAS15 | *E. faecalis* | Activated sludge | Deer Island W.T. Plant | N | KT205598 |
| MAS16 | *E. faecalis* | Activated sludge | Deer Island W.T. Plant | N | KT205599 |
| MAS17 | *E. durans* | Activated sludge | Deer Island W.T. Plant | N | KT205600 |
| MAS18 | *E. hirae* | Activated sludge | Deer Island W.T. Plant | P | KT205601 |
| MAS19 | *E. durans* | Activated sludge | Deer Island W.T. Plant | N | KT205602 |
| MAS2 | *E. durans* | Activated sludge | Deer Island W.T. Plant | N | KT205588 |
| MAS20 | *E. durans* | Activated sludge | Deer Island W.T. Plant | N | KT205603 |
| MAS21 | *E. sanguinicola* | Activated sludge | Deer Island W.T. Plant | N | KT205604 |
| MAS22 | *E. faecalis* | Activated sludge | Deer Island W.T. Plant | P | KT205571 |
| MAS23 | *E. durans* | Activated sludge | Deer Island W.T. Plant | N | KT205605 |
| MAS24 | *E. faecium* | Activated sludge | Deer Island W.T. Plant | N | KT205606 |
| MAS25 | *E. faecalis* | Activated sludge | Deer Island W.T. Plant | N | KT205607 |
| MAS27 | *E. faecalis* | Activated sludge | Deer Island W.T. Plant | N | KT205608 |
| MAS30 | *E. faecalis* | Activated sludge | Deer Island W.T. Plant | P | KT205609 |
| MAS5 | *E. durans* | Activated sludge | Deer Island W.T. Plant | P | KT205589 |
| MAS6 | *E. faecalis* | Activated sludge | Deer Island W.T. Plant | N | KT205590 |
| MAS8 | *E. faecalis* | Activated sludge | Deer Island W.T. Plant | N | KT205591 |
| MAS9 | *E. hirae* | Activated sludge | Deer Island W.T. Plant | N | KT205592 |
| MFG1 | *E. hirae* | Sand | Wollaston Beach, Quincy, MA | P | KT205761 |
| MFG3 | *E. hirae* | Sand | Wollaston Beach, Quincy, MA | P | KT205762 |
| MFG4-1 | *E. hirae* | Sand | Wollaston Beach, Quincy, MA | P | KT205763 |
| MFG4-2 | *E. casseliflavus* | Sand | Wollaston Beach, Quincy, MA | P | KT205764 |
| MFG7 | *E. durans* | Sand | Wollaston Beach, Quincy, MA | P | KT205765 |
| MNCP1B | *E. durans* | Chicken feces | Marshfield, MA | N | KT205633 |
| MNCP2A | *E. faecalis* | Chicken feces | Marshfield, MA | N | KT205634 |
| MNCP2B | *E. faecium* | Chicken feces | Marshfield, MA | N | KT205635 |
| MNCP3A | *E. durans* | Chicken feces | Marshfield, MA | N | KT205636 |
| MNCP4A | *E. durans* | Chicken feces | Marshfield, MA | N | KT205637 |
| MWRA1 | *E. durans* | Activated sludge | Deer Island W.T. Plant | N | KT205651 |
| MWRA10 | *E. durans* | Activated sludge | Deer Island W.T. Plant | N | KT205660 |
| MWRA11 | *E. faecium* | Activated sludge | Deer Island W.T. Plant | N | KT205661 |
| MWRA12 | *E. durans* | Activated sludge | Deer Island W.T. Plant | N | KT205662 |
| MWRA13 | *E. hirae* | Activated sludge | Deer Island W.T. Plant | P | KT205663 |
| MWRA14 | *E. hirae* | Activated sludge | Deer Island W.T. Plant | P | KT205664 |
| MWRA15 | *E. hirae* | Activated sludge | Deer Island W.T. Plant | P | KT205665 |
| MWRA16 | *E. durans* | Activated sludge | Deer Island W.T. Plant | P | KT205666 |
| MWRA17 | *E. hirae* | Activated sludge | Deer Island W.T. Plant | P | KT205667 |
| MWRA18 | *E. hirae* | Activated sludge | Deer Island W.T. Plant | P | KT205668 |
| MWRA19 | *E. hirae* | Activated sludge | Deer Island W.T. Plant | P | KT205669 |
| MWRA2 | *E. hirae* | Activated sludge | Deer Island W.T. Plant | N | KT205652 |
| MWRA20 | *E. hirae* | Activated sludge | Deer Island W.T. Plant | P | KT205670 |
| MWRA21 | *E. durans* | Activated sludge | Deer Island W.T. Plant | N | KT205671 |
| MWRA22 | *E. faecalis* | Activated sludge | Deer Island W.T. Plant | P | KT205672 |
| MWRA23 | *E. hirae* | Activated sludge | Deer Island W.T. Plant | P | KT205673 |
| MWRA24 | *E. hirae* | Activated sludge | Deer Island W.T. Plant | P | KT205674 |
| MWRA25 | *E. durans* | Activated sludge | Deer Island W.T. Plant | N | KT205675 |
| MWRA26 | *E. durans* | Activated sludge | Deer Island W.T. Plant | N | KT205676 |
| MWRA27 | *E. faecalis* | Activated sludge | Deer Island W.T. Plant | N | KT205677 |
| MWRA28 | *E. durans* | Activated sludge | Deer Island W.T. Plant | N | KT205678 |
| MWRA29 | *E. faecium* | Activated sludge | Deer Island W.T. Plant | N | KT205679 |
| MWRA3 | *E. hirae* | Activated sludge | Deer Island W.T. Plant | P | KT205653 |
| MWRA30 | *E. faecalis* | Activated sludge | Deer Island W.T. Plant | N | KT205680 |
| MWRA31 | *E. hirae* | Activated sludge | Deer Island W.T. Plant | P | KT205681 |
| MWRA32 | *E. faecalis* | Activated sludge | Deer Island W.T. Plant | N | KT205682 |
| MWRA33 | *E. durans* | Activated sludge | Deer Island W.T. Plant | N | KT205683 |
| MWRA34 | *E. durans* | Activated sludge | Deer Island W.T. Plant | N | KT205684 |
| MWRA35 | *E. durans* | Activated sludge | Deer Island W.T. Plant | N | KT205685 |
| MWRA36 | *E. faecalis* | Activated sludge | Deer Island W.T. Plant | P | KT205686 |
| MWRA37 | *E. faecalis* | Activated sludge | Deer Island W.T. Plant | P | KT205582 |
| MWRA38 | *E. hirae* | Activated sludge | Deer Island W.T. Plant | P | KT205583 |
| MWRA39 | *E. hirae* | Activated sludge | Deer Island W.T. Plant | N | KT205584 |
| MWRA4 | *E. hirae* | Activated sludge | Deer Island W.T. Plant | P | KT205654 |
| MWRA40 | *E. faecium* | Activated sludge | Deer Island W.T. Plant | N | KT205585 |
| MWRA42 | *E. faecalis* | Activated sludge | Deer Island W.T. Plant | N | KT205620 |
| MWRA43 | *E. hirae* | Activated sludge | Deer Island W.T. Plant | N | KT205621 |
| MWRA44 | *E. faecalis* | Activated sludge | Deer Island W.T. Plant | N | KT205622 |
| MWRA45 | *E. durans* | Activated sludge | Deer Island W.T. Plant | N | KT205623 |
| MWRA46 | *E. hirae* | Activated sludge | Deer Island W.T. Plant | P | KT205624 |
| MWRA47 | *E. durans* | Activated sludge | Deer Island W.T. Plant | N | KT205625 |
| MWRA48 | *E. hirae* | Activated sludge | Deer Island W.T. Plant | N | KT205626 |
| MWRA49 | *E. durans* | Activated sludge | Deer Island W.T. Plant | N | KT205586 |
| MWRA5 | *E. hirae* | Activated sludge | Deer Island W.T. Plant | P | KT205655 |
| MWRA50 | *E. hirae* | Activated sludge | Deer Island W.T. Plant | P | KT205587 |
| MWRA6 | *E. hirae* | Activated sludge | Deer Island W.T. Plant | P | KT205656 |
| MWRA7 | *E. durans* | Activated sludge | Deer Island W.T. Plant | N | KT205657 |
| MWRA8 | *E. hirae* | Activated sludge | Deer Island W.T. Plant | P | KT205658 |
| MWRA9 | *E. faecalis* | Activated sludge | Deer Island W.T. Plant | N | KT205659 |
| NCP1 | *E. durans* | Feline feces | Swampscott, MA | N | KT205617 |
| NCP3 | *E. durans* | Feline feces | Swampscott, MA | N | KT205618 |
| NCP4 | *E. durans* | Feline feces | Swampscott, MA | N | KT205619 |
| NK2 | *E. faecalis* | Unknown environmental | Malden | N | KT205838 |
| NS4F | *E. hirae* | Pigeon feces | North Station (Pigeons) | P | KT205580 |
| NS5C | *E. durans* | Pigeon feces | North Station (Pigeons) | N | KT205581 |
| OG2F | *E. hirae* | Pigeon feces | Oak Grove Station (Pigeons) | N | KT205614 |
| OG6F | *E. faecium* | Pigeon feces | Oak Grove Station (Pigeons) | N | KT205576 |
| PBB2 | *E. hirae* | Canine feces | Unknown | P | KT205638 |
| RM180V | *E. faecalis* | *Spartina alterniflora* | Rowley Marsh, Rowley, MA | N | KT205744 |
| SCP2 | *E. faecalis* | Topsoil | Lake Quannapowitt, Wakefield | P | KT205569 |
| WB1c | *E. faecalis* | Toilet swab | UMass Boston, MA | N | KT205837 |
| WBAM1 | *E. faecalis* | Water | Whitman Pond, Weymouth | N | KT205836 |
| WY | *E. hirae* | Topsoil | Boston, MA | N | KT205570 |
