# Supplementary material for: Incidence of Type II CRISPR1-Cas Systems in Enterococcus Is Species-Dependent
Source: PLoS One. 2015 Nov 24;10(11):e0143544. doi: 10.1371/journal.pone.0143544 (PMC4658022; doi:10.1371/journal.pone.0143544)
Supplement: S1 Text — (DOCX) [file pone.0143544.s003.docx]

**Supplemental Methods**

The *E. faecalis* OG1RF *cas1* sequence (accession CP002621.1) was used as the query sequence for a BLASTn search of the nucleotide collection (nr/nt) database, limiting results to Bacteria (taxid: 2). Results were then further limited to include only enterococcal species, the target of this study (taxid: 1350). Sixteen *Enterococcus* strains containing putative *cas1* homologs were found (Figure A).


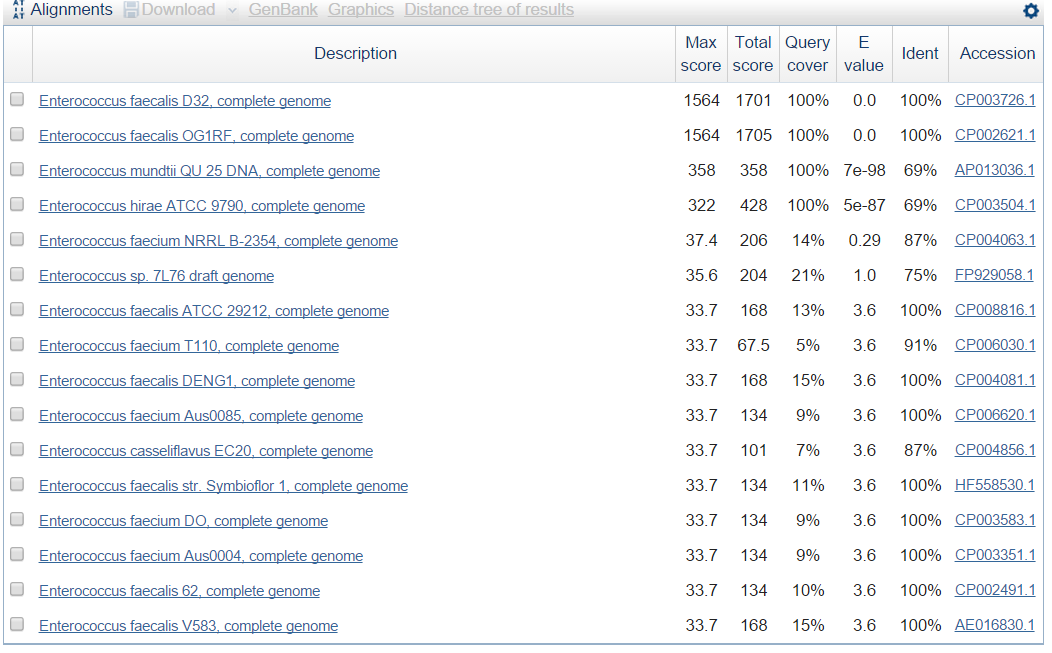


**Figure A: Enterococcus cas1 homologs.** Putative homologs were located by BLASTn search of NCBI nucleotide collection (nr/nt) database, using the E. faecalis OG1RF cas1 gene sequence as query.

Thirteen of these strains are in CRISPRdb and were analyzed previously. The remaining three (*E. faecalis* ATCC 29212, *E. faecalis* DENG1, and *E. faecium* T110) were examined using CRISPRfinder (Table A).

**Table A: CRISPRs and cas genes in Enterococcus genomes.** CRISPRfinder and CRISPRdb examination of Enterococcus genomes containing putative cas1 homologs identified by BLASTn search. “Questionable structures” are defined by CRISPRfinder/CRISPRdb as either small CRISPRs containing fewer than 3 repeats, or structures where the repeats are not 100% identical. Shaded rows indicate BLASTn results with query cover < 25% and/or E-values > 1.

|  | Accession | Confirmed CRISPRs | Questionable Structures | Referenced *cas* genes in Genbank |
| --- | --- | --- | --- | --- |
| *Enterococcus faecalis* D32 | CP003726.1 | 2 | 0 | *cas1*, *cas2*, *cas9*, *csn2* |
| *Enterococcus faecalis* OG1RF | CP002621.1 | 2 | 0 | *cas1*, *cas2*, *cas9*, *csn2* |
| *Enterococcus mundtii* QU 25 | AP013036.1 | 0 | 3 | *csn2* |
| *Enterococcus hirae* ATCC 9790 | CP003504.1 | 1 | 0 | *cas1*, *cas2*, *cas9*, *csn2* |
| *Enterococcus faecium* NRRL B-2354 | CP004063.1 | 0 | 1 | - |
| *Enterococcus* sp. 7L76 | FP929058.1 | 1 | 1 | *cas2* |
| *Enterococcus faecalis* ATCC 29212 | CP008816.1 | 1 | 1 | - |
| *Enterococcus faecium* T110 | CP006030.1 | 0 | 2 | *cmr6* |
| *Enterococcus faecalis* DENG1 | CP004081.1 | 1 | 1 | - |
| *Enterococcus faecium* Aus0085 | CP006620.1 | 0 | 0 | - |
| *Enterococcus casseliflavus* EC20 | CP004856.1 | 0 | 0 | - |
| *Enterococcus faecalis* str. Symbioflor1 | HF558530.1 | 1 | 1 | - |
| *Enterococcus faecium* DO | CP003583.1 | 0 | 0 | - |
| *Enterococcus faecium* Aus0004 | CP003351.1 | 0 | 0 | - |
| *Enterococcus faecalis* 62 | CP002491.1 | 1 | 2 | - |
| *Enterococcus faecalis* V583 | AE016830.1 | 0 | 2 | - |

Low query cover and high E-values for 12 of the 16 BLASTn results suggest that these may not be Type II-A CRISPR1-*cas1* homologs. *Enterococcus faecalis* V583 has been previously characterized as not containing an active CRISPR-Cas system ([1](#_ENREF_1)). Matches from enterococcal genomes not characterized in CRISPRdb or CRISPRfinder as containing confirmed CRISPRs may also be false positives. Additionally, these genes may belong to different CRISPR types or subtypes. Type III CRISPR-Cas components have recently been identified in *Enterococcus* species ([2](#_ENREF_2)). The putative *E. faecium* T110 *cas1* homolog has only 5% query cover, and an E-value of 3.6. Further examination of this strain in CRISPRfinder reveals that a referenced *cas* gene is *cmr6*, a Type III-B gene ([3](#_ENREF_3)). These sequences were therefore not used to design the *cas1* primers. The primer set utilized in this study will not amplify Type I or Type III *cas1*, nor the Type II-A CRISPR3-*cas1*.

The sequence for the putative *E. mundtii* QU 25 *cas1* gene was added to GenBank after the primers used in this study were designed, and the E-value of the match (7e-98) suggests that it may be a Type II-A homolog. Multiple sequence alignment was performed using MUSCLE as previously described, to include the *E. mundtii* sequence with the *cas1* homologs used for primer design. It is noted that the *cas1* primers in this study would not amplify the putative *cas1* gene *in silico* in *E. mundtii* QU 25 (Figure B).


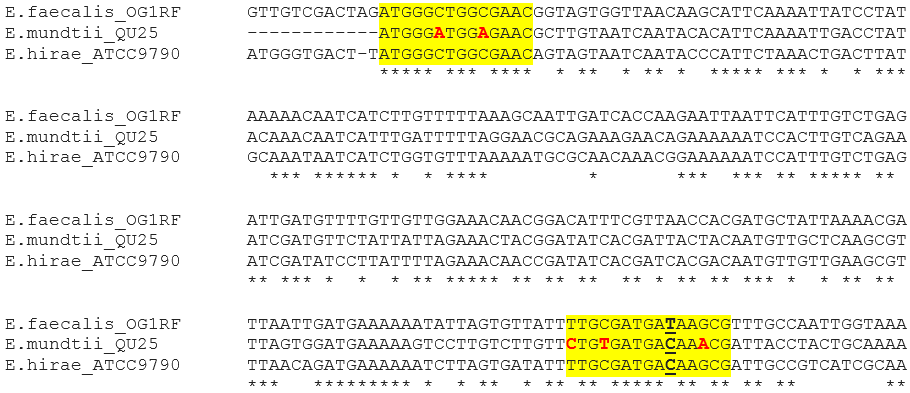


**Figure B: Multiple sequence alignment of cas1 homologs, showing primer specificity.** Yellow highlighted regions indicate the primer target sequences. Red bold-type bases indicate discrepancies between the primers designed using E. hirae ATCC 9790 and E. faecalis OG1RF cas1 sequences, and the E. mundtii QU25 cas1 sequence in GenBank. Underlined bold-type indicates the degeneracy designed into the existing cas1 primers; this discrepancy does not affect primer specificity.

References

1. Palmer KL, Gilmore MS. Multidrug-resistant enterococci lack CRISPR-cas. MBio. 2010;1(4).

2. Duerkop BA, Palmer KL, Horsburgh MJ. Enterococcal Bacteriophages and Genome Defense. In: Gilmore MS, Clewell DB, Ike Y, Shankar N, editors. Enterococci: From Commensals to Leading Causes of Drug Resistant Infection. Boston2014.

3. Makarova KS, Haft DH, Barrangou R, Brouns SJ, Charpentier E, Horvath P, et al. Evolution and classification of the CRISPR-Cas systems. Nat Rev Microbiol. 2011;9(6):467-77.
